# Cytochrome P450 26A1 regulates the clusters and killing activity of NK cells during the peri‐implantation period

**DOI:** 10.1111/jcmm.17269

**Published:** 2022-03-16

**Authors:** Dan‐Dan Li, Wen‐Heng Ji, Dan‐Ping Wei, Ai‐Qin Gu, Zhi‐Hui Song, Wen‐Ning Fang, Chao‐Yang Meng, Ying Yang, Jing‐Pian Peng

**Affiliations:** ^1^ State Key Laboratory of Stem Cell and Reproductive Biology Institute of Zoology Chinese Academy of Sciences Beijing China; ^2^ University of Chinese Academy of Sciences Beijing China

**Keywords:** CYP26A1, cytotoxicity assay, NK cells, scRNA‐seq

## Abstract

Cytochrome P450 26A1 (CYP26A1) plays a vital role in early pregnancy in mice. Our previous studies have found that CYP26A1 affects embryo implantation by modulating natural killer (NK) cells, and that there is a novel population of CYP26A1^+^ NK cells in the uteri of pregnant mice. The aim of this study was to investigate the effects of CYP26A1 on the subsets and killing activity of NK cells. Through single‐cell RNA sequencing (scRNA‐seq), we identified four NK cell subsets in the uterus, namely, conventional NK (cNK), tissue‐resident NK (trNK) 1 and 2, and proliferating trNK (trNKp). The two most variable subpopulations after uterine knockdown of CYP26A1 were trNKp and trNK2 cells. CYP26A1 knockdown significantly downregulated the expression of the NK cell function‐related genes *Cd44*, *Cd160*, *Vegfc*, and *Slamf6* in trNK2 cells, and *Klra17* and *Ogn* in trNKp cells. Both RNA‐seq and cytotoxicity assays confirmed that CYP26A1^+^ NK cells had low cytotoxicity. These results indicate that CYP26A1 may affect the immune microenvironment at the maternal‐foetal interface by regulating the activity of NK cells.

## 1 INTRODUCTION

Pregnancy is a unique biological event in which a mother peacefully coexists with a semi‐allogeneic foetus. During the first trimester of pregnancy, leukocytes account for ~30%–40% of cells in the human decidua. Natural killer (NK) cells make up ~70% of decidual leukocytes.^1^, ^2^ Uterine NK cells in mice are defined as CD45^+^Lin^−^NK1.1^+^NKp46^+^ during pregnancy. Two different functional NK cell populations can be identified by their ability to bind to the lectin *Dolichos biflorus* agglutinin (DBA) in mice.^3^ DBA^+^ NK cells lack the expression of CD49b but overwhelmingly express angiogenic factors, whereas *Ifng* expression prevails among the DBA^−^ NK cells which are CD49b positive.^4^, ^5^ Tian et al.^6^ define CD49a as a specific cell surface marker of NK cells residing in tissue. Based on this, murine NK cells are classified into CD49a^−^ conventional NK (cNK) cells and CD49a^+^ tissue‐resident NK (trNK) cells.^7^, ^8^ trNK cells are dominant during early gestation, most abundant on gestation day (GD)5.5, and decrease in number as pregnancy progresses. After placenta formation, cNK cells expand rapidly and dominate during late pregnancy.^9^

NK cells, the major leukocyte population at the maternal‐foetal interface, play a vital role in embryo implantation, trophoblast invasion and spiral artery remodeling during pregnancy.^10^, ^11^ NK cell exhaustion in pregnant mice leads to a reduction in the number of implantation sites, barren vascular remodelling and an increase in the embryo resorption rate.^12^, ^13^ In humans, NK cells infiltrate the decidua and locate nearby extravillous trophoblast cells (EVTs) during early pregnancy. Certain combinations of human leukocyte antigen C expressed by EVTs and killer immunoglobulin‐like receptors on decidual NK (dNK) cells contribute to increased risk of preeclampsia. This is due to excessive inhibition of dNK cells, which results in poor trophoblast invasion.^14^ Conversely, unduly active uterine NK cells are closely related to pregnancy failure.^15^, ^16^

Cytochrome P450 26A1 (CYP26A1), a member of the cytochrome P450 superfamily, is a monooxygenase that catalyses the metabolism of all‐trans‐retinoic acid (at‐RA).^17^ *Cyp26a1*‐null mice die mid‐pregnancy or during terminal pregnancy and exhibit numerous crucial morphogenetic defects, such as aberrant hindbrain patterning and vertebral identity.^18^ Our previous work has found that CYP26A1 shows a peculiar temporal and spatial expression pattern in mice and rats during the peri‐implantation period.^19^, ^20^ In Cyp26a1‐MO‐treated and pCR3.1‐Cyp26a1 plasmid‐immunized mice, the number of implantation sites significantly decreases and the proportion of NK cells, dendritic cells (DCs) and macrophages changes dramatically.^21^, ^22^, ^23^ dNK1 cells that highly express *CYP26A1* have been identified during early gestation in humans.^24^ Previous studies in our laboratory have also found a novel population of CYP26A1^+^ NK cells in the uterus.^25^ However, little is known about the underlying mechanisms involved, especially the effect of CYP26A1 on NK cell immune activity.

In this study, single‐cell RNA sequencing (scRNA‐seq) analysis revealed four major NK cell subsets in the uterus. CYP26A1 knockdown had no effect on the clusters of NK cells but it affected the proportion of NK cell subsets and downregulated the expression of genes associated with immune activity and cytokines in NK cells. Further experiments indicated that CYP26A1^+^ NK cells had low killing activity. Hence, CYP26A1 may influence the immune microenvironment at the maternal‐foetal interface by modulating the activity of NK cells.

## 2 MATERIALS AND METHODS

### 2.1 Mice

BALB/c mice, aged 8–10 weeks, were purchased from Vital River Laboratory Animal Technology Co. Ltd. (Beijing). The mice were housed in the animal care facility of the Institute of Zoology, Chinese Academy of Sciences, according to the institutional guidelines for the care and use of laboratory animals. Male mice cohabited with females at a ratio of 1:1. The day when the vaginal plug was detected was recorded as the first day of pregnancy (GD1).

### 2.2 Preparation of single‐cell suspension and flow cytometry analysis

The uterus and spleen were cut apart and minced into small fragments. Splenic tissue fragments were placed in PBS containing 2% FBS. Uterine fragments were placed in 1640 medium containing 200 U/ml hyaluronidase (H3506, Sigma‐Aldrich), 1 mg/ml collagenase type IV (C5138, Sigma‐Aldrich) and 8% FBS and then incubated at 37°C for 30 min, as previously described but with minor changes.^26^ After digestion, uterus cells were centrifuged and incubated in 1640 medium containing 2% FBS at 37°C for 15 min. The uterus and spleen cells were then filtered through a 37 μm nylon mesh. After centrifugation, the cells were re‐suspended in PBS containing 2% FBS for staining. The cell suspensions were blocked with anti‐mouse CD16/CD32 (14–0161–81, Invitrogen) for 10 min and then incubated with CYP26A1 primary antibody and fluorescently labelled antibodies for 30 min respectively. The antibodies used for flow cytometry analysis were as follows: anti‐CD45 PerCP‐Cyanine5.5 (45–0451–80, Invitrogen), anti‐CD45 APC/Cyanine7 (103115, BioLegend), anti‐CD45 Brilliant Violet 510 (103138, BioLegend), anti‐CD3ε FITC (100305, BioLegend), anti‐CD3e APC (17–0031–81, Invitrogen), anti‐CD3e PE (12–0031–81, Invitrogen), anti‐CD122 PE (123209, BioLegend), anti‐CD122 Brilliant Violet 421 (566301, BD Biosciences), anti‐CD122 APC (17–1222–80, Invitrogen), anti‐CD49b Pacific Blue (108918, BioLegend), DBA FITC (L32474, Invitrogen), anti‐CYP26A1 (PA5‐24602, Invitrogen), anti‐rabbit IgG Alexa Fluor 488 (A‐21206, Invitrogen) and anti‐rabbit IgG Brilliant Violet 421 (406410, BioLegend). After staining, the cells were washed and re‐suspended in PBS containing 2% FBS for analysis with a BD LSR Fortessa or BD AriaFusion (BD Biosciences) instrument. The data were analysed using FlowJo X 10.0.7 R2.

### 2.3 Morpholino antisense oligonucleotide (MO) knockdown mice

MOs were administered via intrauterine injection, as previously described but with minor modifications.^19^, ^27^ Cyp26a1‐MO (5′‐CATGGCACGCCCCCTCCCGCGC‐3′) and Random Control‐MO (5′‐25‐N‐3′) were purchased from Gene Tools, LLC (Philomath, OR 97370, USA). MOs at a final concentration of 4 mM were prepared with sterile distilled water and stored at 25°C in a humid environment. At 8:30 AM, 30 nmol Cyp26a1‐MO or Random Control‐MO solution was injected into the uterine horn of anaesthetized GD4 mice. On GD6, the sacrificed mice were dissected and the uteri were collected for further analysis.

### 2.4 Western blotting

Mouse uterine tissue was ground into powder in liquid nitrogen and added to RIPA Lysis Buffer (CW2334S, Cwbio) containing 1 mM PMSF (78830, Sigma). Total protein was extracted following the instructions of the RIPA Lysis Buffer kit. The Bicinchoninic Acid Protein Assay Kit (23227, Pierce) was used to detect the protein concentration. Proteins were separated using 10% SDS‐PAGE and then transferred from the gel onto a nitrocellulose membrane (66485, Pall). The membrane was blocked with 5% skimmed milk and then incubated overnight with primary antibodies at 4°C. After washing thoroughly with TBST solution, the membranes were incubated with HRP‐coupled secondary antibodies at room temperature for 1 h and then visualized using a chemiluminescence imaging system (MiniChemi 610, Sagecreation). The data were analysed using ImageJ software. The primary and secondary antibodies used for Western blotting included anti‐CYP26A1 (ab151968, 1:1000, Abcam), anti‐GAPDH (2118, 1:1000, Cell Signaling Technology) and goat anti‐rabbit IgG (H + L) HRP (31460, 1:10,000, Thermo Fisher).

### 2.5 scRNA‐seq and analysis

Viable CD45^+^CD3^−^CD122^+^ cells were sorted from the uteri of five Cyp26a1‐MO‐treated mice and two controls on GD6. An estimated 10,000 cells per library were captured using a Chromium Single Cell 3′ kit v3.1 (10x Genomics). Libraries containing read 1 (28 bp: 16 bp 10x cell barcode and 12 bp UMI), read 2 (91 bp cDNA fragment) and i7 index read (8 bp sample index) were sequenced on an Illumina NovaSeq 6000 sequencer.

Cell Ranger software was used to process raw sequencing data, and the mm10 mouse reference genome assembly was used as the reference index. Cells were removed when one of the following conditions was met: (i) the number of detected genes was more than 8000 or less than 300; (ii) the number of UMIs was less than 500; and (iii) the mitochondrial gene expression level was higher than 20%. In addition, genes expressed in more than three cells were kept.

Raw count matrices, obtained by reading the output of the Cell Ranger pipeline, were converted into Seurat objects, which were merged together into a single Seurat object for downstream analysis according to Seurat (version 4.0.6) tutorials.^28^ These analyses, including normalization, variance stabilization, sample integration using shared hypervariable genes, cell clustering based on top principal components, differential expression analysis and visualization, were carried out following the standard process of Seurat. To characterize the functional properties of four NK cell subsets, KEGG analysis of differentially expressed genes (Absolute log_2_Fold change > 0.5 and adjusted *p* < 0.05) among these NK cell subsets was performed using the R package clusterProfiler (version 4.2.0).^29^ clusterProfiler was also used for GO analysis of differentially expressed genes (adjusted *p* < 0.05; |log_2_Fold change| > 0.5) among Cyp26a1‐MO treated mice and controls.

### 2.6 Immunocytofluorescence

CD45^+^CD3^−^CD122^+^CYP26A1^+^ and CD45^+^CD3^−^CD122^+^CYP26A1^−^ NK cells were isolated from GD5 mice by flow cytometry. After centrifugal re‐suspension, cells were incubated with DAPI (2 μg/ml) at room temperature for 15 min. After washing, the cell suspensions were dropped onto a glass slide and covered with a cover slip for imaging. Images were captured using a Zeiss LSM 780 confocal microscope and analysed with ZEN software.

### 2.7 NK cell‐mediated cytotoxicity assay

Target YAC‐1 cells were labelled with carboxyfluorescein succinimidyl ester (CFSE; C1031, Beyotime) at a final concentration of 5 μM. They were then co‐cultured with effector NK cells at an effector‐to‐target cell (E:T) ratio of 2:1 or 0:1 for 4 h at 37°C under 5% CO_2_. After 4 h, the samples were stained with Helix NP NIR (425301, BioLegend) and Annexin V‐PE (640908, BioLegend) for 10 min. Fluorescence‐activated cell sorting (FACS) analysis was performed immediately with a BD LSR Fortessa Cell Analyzer (BD Biosciences). The Incucyte S3 Live‐Cell Analysis Instrument (Sartorius AG) was used to monitor the killing activity of NK cells. Propidium iodide (C1052, Beyotime) was used to mark dead cells.

### 2.8 RNA‐seq and analysis

Uterine CD45^+^CD3^−^CD122^+^CYP26A1^+^ and CD45^+^CD3^−^CD122^+^CYP26A1^−^ NK cells were sorted from GD5 mice using flow cytometry. Total RNA was extracted using TRIzol^®^ reagent (15596, Ambion). A NanoDrop 2000 micro‐spectrophotometer, an Agilent 2100 bioanalyzer, and an Agilent RNA 6000 Pico Kit were used to evaluate the purity, concentration and integrity of RNA samples separately. Transcriptome libraries were constructed using the SMART‐seq2 method and sequenced using the Illumina NovaSeq 6000 platform. The mm10 mouse genome assembly was used as the reference genome, and HISAT2 (tophat) was used to align raw sequencing reads with the default parameters. Counts for protein‐coding genes were acquired using HTSeq (version 0.6.0). Differentially expressed genes (adjusted *p* < 0.05; |Fold change| > 2) were determined with DESeq2 (version 1.30.1) and further gene set enrichment analysis (GSEA) was performed using clusterProfiler (version 4.2.0).^29^

### 2.9 RNA extraction and quantitative real‐time PCR (qPCR)

Total RNA was extracted from NK cells with TRIzol^®^ reagent (15596018, Invitrogen) and reverse‐transcribed into cDNA with the M‐MLV reverse transcriptase reaction system (M1705, Promega) in accordance with the manufacturer's instructions. qPCR was performed with 2x UltraSYBR Mixtures (CW0957 M, Cwbio) on a LightCycler 480 II instrument (Roche). Expression levels of target genes were normalized to *Gapdh* expression. The 2^−ΔΔCt^ (cycle threshold, Ct) method was used to calculate the relative abundance of gene transcripts. The primers used for the qPCR experiments are listed in Table 1.

**TABLE 1:** List of mouse primers for qPCR analysis

Gene	Sequences (5′−3′)	Product length
*Gapdh*	Forward: AGGTCGGTGTGAACGGATTTG Reverse: TGTAGACCATGTAGTTGAGGTCA	123 bp
*Tlr8*	Forward: GCCAAACAACAGCACCCAAA Reverse: AAAGACTCAGGCAACCCAGCA	140 bp
*Fcgr1*	Forward: AGCATCCCAGAGGCCAGTTTT Reverse: AGGCCAGGGGTTCTCCTTCT	154 bp
*Siglece*	Forward: CCCAGATGCCCTCAAGGTCT Reverse: GTAGTGTCCTGTTGCCCCCA	186 bp
*Vav2*	Forward: ACACTGCCTACCCGTGGTTT Reverse: CCTCTGCTGGACGCTCTCTG	108 bp
*Tnf*	Forward: GCCTCTTCTCATTCCTGCTTG Reverse: CTGATGAGAGGGAGGCCATT	115 bp
*Klrk1*	Forward: ACGACCTCAAGCCAGCAAAG Reverse: AGCAAGGACTCGAACAACGAAC	158 bp
*Gzmb*	Forward: CATCATAAAAGCAGAGAGGGGGT Reverse: CGAGAGTGGGGCTTGACTTC	220 bp
*Klre1*	Forward: ACCTGTAACCCGTTCTACCCT Reverse: CATGGCACCGAGCACACTC	220 bp
*Ifng*	Forward: ACAGCAAGGCGAAAAAGGATG Reverse: TGGTGGACCACTCGGATGA	106 bp
*Ikzf3*	Forward: GCTGCAAGTGTGGAGGCAAG Reverse: CCGGGTCTGCATCATCTCGT	201 bp

### 2.10 Statistical analysis

Data were analysed with GraphPad Prism (version 9.0.0) software. The results were expressed as means ± SEM. The paired or unpaired two‐tailed t‐test was used to evaluate the differences. Statistically significant differences were defined as *p* values < 0.05.

## 3 RESULTS

### 3.1 Analysis of NK cell clustering via scRNA‐seq

**FIGURE 1:**
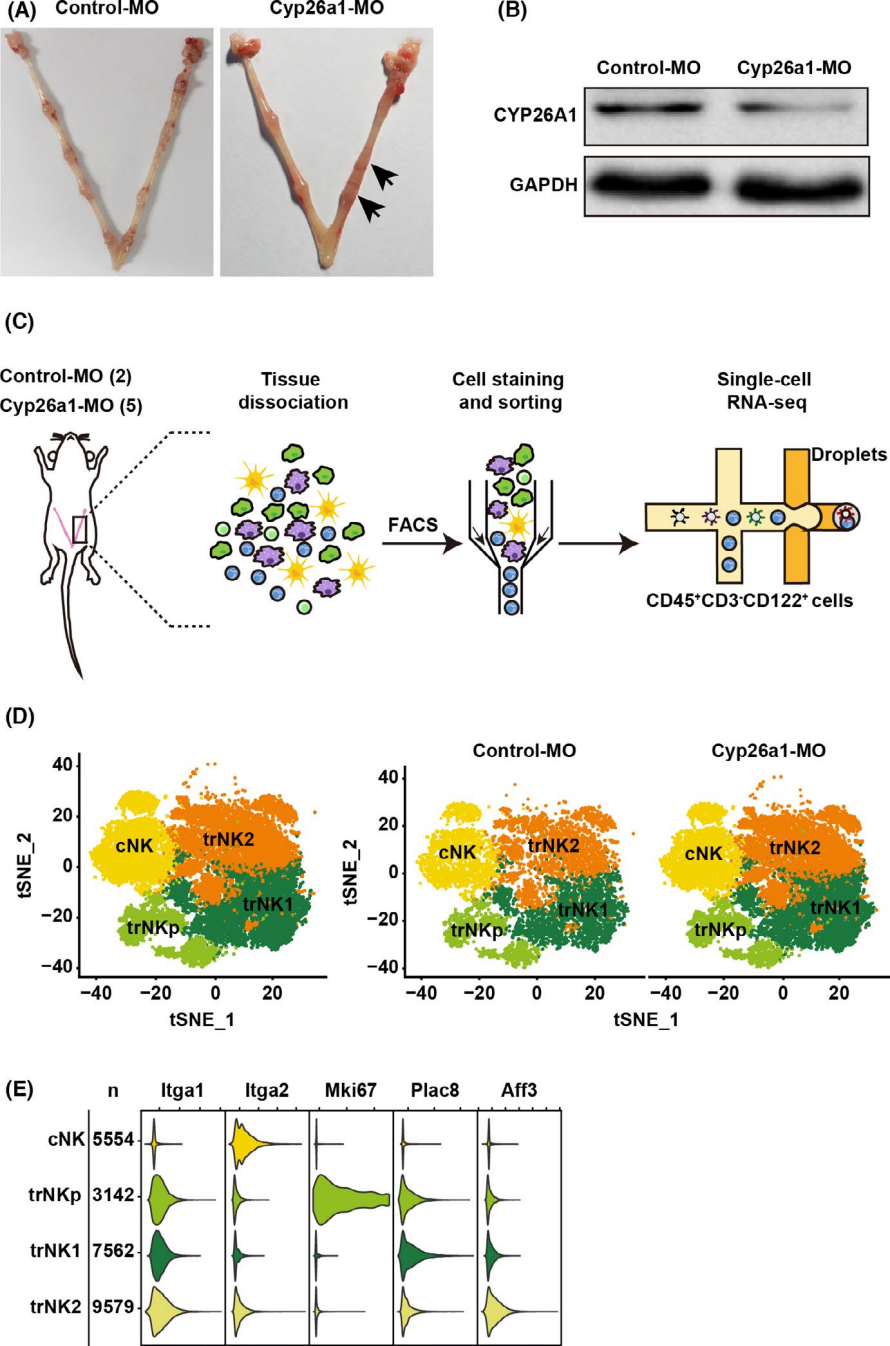
Atlas of NK cells in Cyp26a1‐MO‐treated mice. (A) Representative macroscopic views of uteri from Control‐MO and Cyp26a1‐MO treated mice. Arrows represent abnormal implantation sites. (B) CYP26A1 expression in uterus from Cyp26a1‐MO knockdown mouse model analysed by western blotting. (C) Workflow for single‐cell transcriptome profiling of NK cells from Control‐MO or Cyp26a1‐MO‐treated mouse uterus. CD45^+^CD3^−^CD122^+^ cells were enriched by FACS and then used for scRNA‐seq. (D) t‐SNE plots of NK cells, showing four main clusters for Cyp26a1‐MO treatment and control‐MO mice. Colours indicate different cell clusters. (E) Violin plots displaying marker genes (top row) for four main NK cell subsets. The first two columns represent the names of the NK cell clusters and the corresponding numbers of cells

To investigate the effects of CYP26A1 on NK cell populations and immune activity, we established a CYP26A1 knockdown mouse model. Briefly, Cyp26a1‐MO was injected into the uterine horn of pregnant mice on GD4, and the mice were sacrificed on GD6 for flow cytometry analysis. Cyp26a1‐MO treatment mice displayed a lower protein expression level of CYP26A1 in the uterus and obviously abnormal embryo implantation compared with the control group (Figure 1A,B).

We sorted uterine CD45^+^CD3^−^CD122^+^ cells (total NK cells) obtained from the CYP26A1 knockdown mouse model (five among Cyp26a1‐MO treatment mice and two among Control‐MO mice) and then performed scRNA‐seq using the 10x platform (Figure 1C). After filtering out the low‐quality cells, we finally acquired the transcriptomes of 25,837 single cells, including 17,538 cells derived from mice with CYP26A1 knockdown and 8,299 cells from controls.

Seurat was used for graph‐based clustering, and four NK cell subsets (cNK, trNKp, trNK1 and trNK2) were identified based on known and cluster‐specific marker genes (Figure 1D).^9^, ^30^ Based on the expression of *Cd49a* (also known as *Itga1*), the tissue‐resident marker, uterine NK cells were classified into CD49a^−^ cNK and CD49a^+^ trNK cells. cNK cells highly expressed *Cd49b* (also known as *Itga2*). trNKp cells exhibited strong expression of *Mki67*, indicating active proliferative capacity. The defining marker of trNK1 cells was *Plac8*. trNK2 cells showed the highest expression of *Cd49b* among the three trNK cell subsets and were characterized by *Aff3* (Figure 1E). CYP26A1 knockdown had no effect on the clustering of uterine NK cells.

### 3.2 CYP26A1 affected NK cell proportion and activity

**FIGURE 2:**
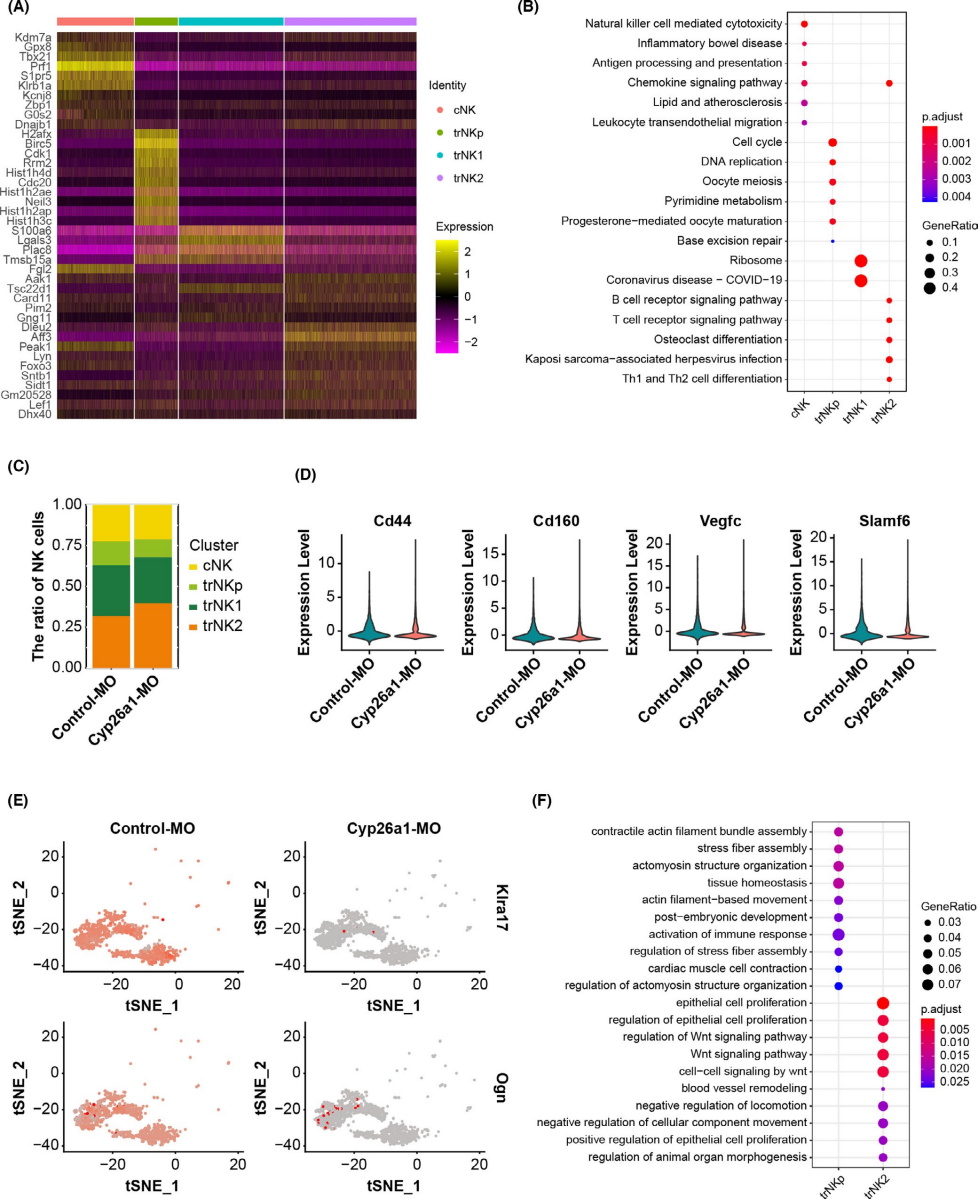
CYP26A1 knockdown affected NK cell proportion and downregulated the expression of cytokines and immune activity‐related genes. (A) Heatmap indicating expression levels of top 10 marker genes for four NK cell subsets. (B) Results of KEGG performed using differentially expressed genes (adjusted *p* < 0.05; |log_2_Fold change| > 0.5) in four NK subsets to illustrate their function. (C) Percentages of cNK, trNKp, trNK1, and trNK2 cell subpopulations among NK cells from Cyp26a1‐MO knockdown and control mice. (D) Violin plots comparing expression levels of *Cd44*, *Cd160*, *Vegfc*, and *Slamf6* in trNK2 cells from Cyp26a1‐MO treatment mice and controls. (E) t‐SNE plots showing expression levels of *Klra17* and *Ogn* in trNKp cell subset from Cyp26a1‐MO and control‐MO mice. (F) GO analysis of differentially expressed genes (adjusted *p* < 0.05; |log_2_Fold change| > 0.5) between Cyp26a1‐MO‐treated mice and controls, assessed for trNKp and trNK2 cells. GeneRatio is the ratio of the number of differentially expressed genes enriched in each GO term to the total number of differentially expressed genes; *p*.adjust is the adjusted *p*‐value. The colour and size of the dots represent *p*.adjust and GeneRatio respectively

As mentioned above, we defined four main NK cell subsets: cNK, trNKp, trNK1 and trNK2. To determine the functional characteristics of these subsets, markers were found for each cluster using Seurat; the expression levels of the top 10 marker genes in each subset are presented in Figure 2A. KEGG pathway analysis was performed using the differentially expressed marker genes (adjusted *p* < 0.05; |log2Fold change| > 0.5) for each subset (Figure 2B). As shown, cNK cells strongly expressed *Prf1* and *Klrb1a* and upregulated the NK cell‐mediated cytotoxicity pathway, demonstrating their significant cytotoxicity. trNKp cells highly expressed *Cdk1* and *Cdc20* in relation to the cell cycle, exhibiting a strong proliferative status. trNK1 cells had an elevated expression level of *Lgals3* and showed active ribosome activity. The Th1 and Th2 cell differentiation pathway was enriched in trNK2 cells.

We first focused on the proportional divergence of the four NK cell subsets in controls and Cyp26a1‐MO treatment mice. The number of trNKp cells was lower in Cyp26a1‐MO‐injected mice, whereas that of trNK2 cells was higher; there were no apparent differences in the proportions of cNK and trNK1 cells (Figure 2C). To further investigate the effect of CYP26A1 knockdown on NK cell function, we determined the differentially expressed genes of trNKp as well as trNK2 cells in Cyp26a1‐MO‐treated mice and controls and performed GO analysis (Figure 2F). CYP26A1 knockdown significantly downregulated the expression of the NK cell‐activating markers *Cd44* and *Cd160*, activating receptor *Slamf6* and vascular endothelial growth factor C (*Vegfc)* in trNK2 cells (Figure 2D). The pathways including epithelial cell proliferation and blood vessel remodelling were enriched in trNK2 cells (Figure 2F). trNKp cells showed significantly downregulated expression levels of the inhibitory receptor *Klra17* and the growth‐promoting factor *Ogn* after CYP26A1 knockdown (Figure 2E). The activation of immune response pathway was enriched in trNKp cells (Figure 2F).

### 3.3 CYP26A1^+^ NK cells underwent dynamic changes

**FIGURE 3:**
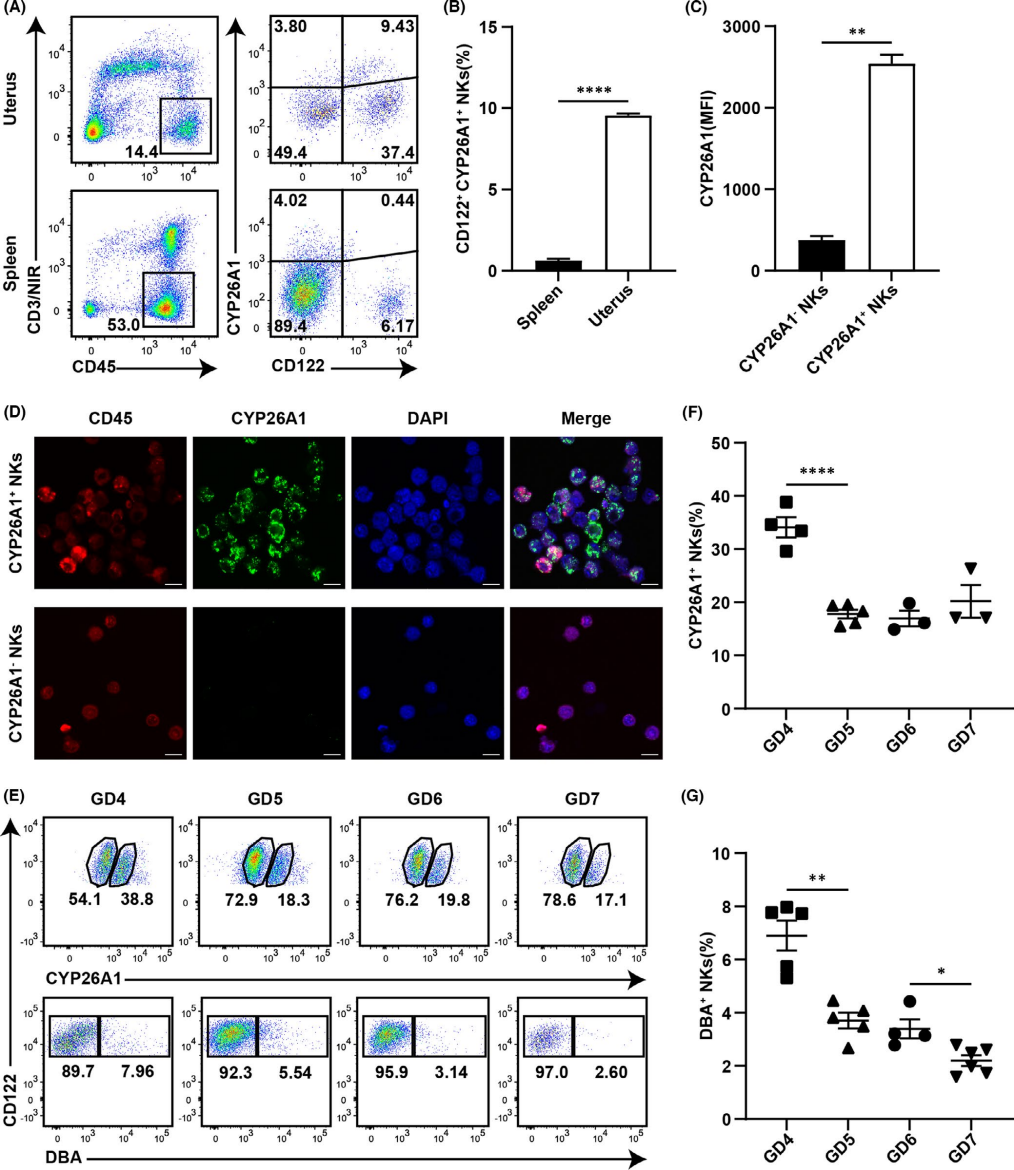
CYP26A1 expression in NK cells and dynamic changes in uterine CYP26A1^+^ NK cells in mice during peri‐implantation period. (A) Representative flow cytometry gating strategy for uterine and splenic cells from mice on GD6. The CD45^+^CD3/NIR^−^ gate represents live leukocytes eliminating T cells. CD122^+^CYP26A1^+^ and CD122^+^CYP26A1^−^ cells typify CYP26A1^+^ and CYP26A1^−^ NK cells, respectively. (B) Statistical calculations of percentages of CD122^+^CYP26A1^+^ cells among CD45^+^CD3/NIR^−^ cells from mouse spleen and uterus. Unpaired t‐test. (C) Median fluorescence intensity (MFI) of CYP26A1 from CD122^+^CYP26A1^−^ and CD122^+^CYP26A1^+^ uterine cells. Paired t test. (D) Results of confocal microscopy to detect CYP26A1 expression in sorted CD45^+^CD3^−^CD122^+^CYP26A1^−^ and CD45^+^CD3^−^CD122^+^CYP26A1^+^ cells in uterus of GD5 mouse. Scale bar: 10 µm. (E) Representative flow cytometry analysis of percentages of CYP26A1^+^ and DBA^+^ cells in CD3^−^CD122^+^ uterine NK cells during peri‐implantation period in mice. Data summary of percentages of (F) CYP26A1^+^ and (G) DBA^+^ NK cells. NKs, NK cells. Data represent means ± SEM of at least three independent experiments. *p < 0.05, **p < 0.01, ****p < 0.0001

To verify the expression of CYP26A1 in NK cells, we employed flow cytometry to detect the proportions of CYP26A1^+^ NK cells isolated from mouse spleens and uteri on GD6. Among mouse uterine cells, approximately 9.5% of CD45^+^CD3^−^ cells were CD122^+^CYP26A1^+^ cells, whereas only 0.6% spleen cells were CD122^+^CYP26A1^+^ cells (Figure 3A,B). Flow cytometry analysis indicated that CYP26A1^+^ NK cells had a higher median fluorescence intensity (MFI) value for CYP26A1 than CYP26A1^−^ cells (Figure 3C). Live‐cell immunofluorescence also showed that CYP26A1 was only present on CYP26A1^+^ NK cells and was primarily located on the cytoplasmic membrane (Figure 3D). These data demonstrated that there was a specific CYP26A1^+^ NK cell subset in the uteri of pregnant mice.

Before investigating the impact of CYP26A1^+^ NK cells on early pregnancy, we detected the changes in the trend of their proportion during the peri‐implantation period. DBA, which has a high affinity for glycoconjugates containing *N*‐acetyl‐d‐galactosamine in the terminal position, is known as the specific marker for mouse uterine NK cells.^3^ Flow cytometry was used to analyse the proportions of CYP26A1^+^ and DBA^+^ cells among NK cells marked as CD45^+^CD3^−^CD122^+^ in mice from GD4 to GD7 (Figure 3E). The proportion of CYP26A1^+^ cells was highest (34.10%) on GD4, decreased dramatically on GD5 (*p* < 0.0001), and was maintained at a relatively stable level (~20%) from GD5 to GD7 (Figure 3F). The proportion of DBA^+^ cells peaked on GD4 (6.90%), decreased significantly from GD4 to GD5 (*p* = 0.0011), and showed a further significant reduction from GD6 to GD7 (*p* = 0.0138) (Figure 3G).

### 3.4 Killing activity of CYP26A1^+^ NK cells

**FIGURE 4:**
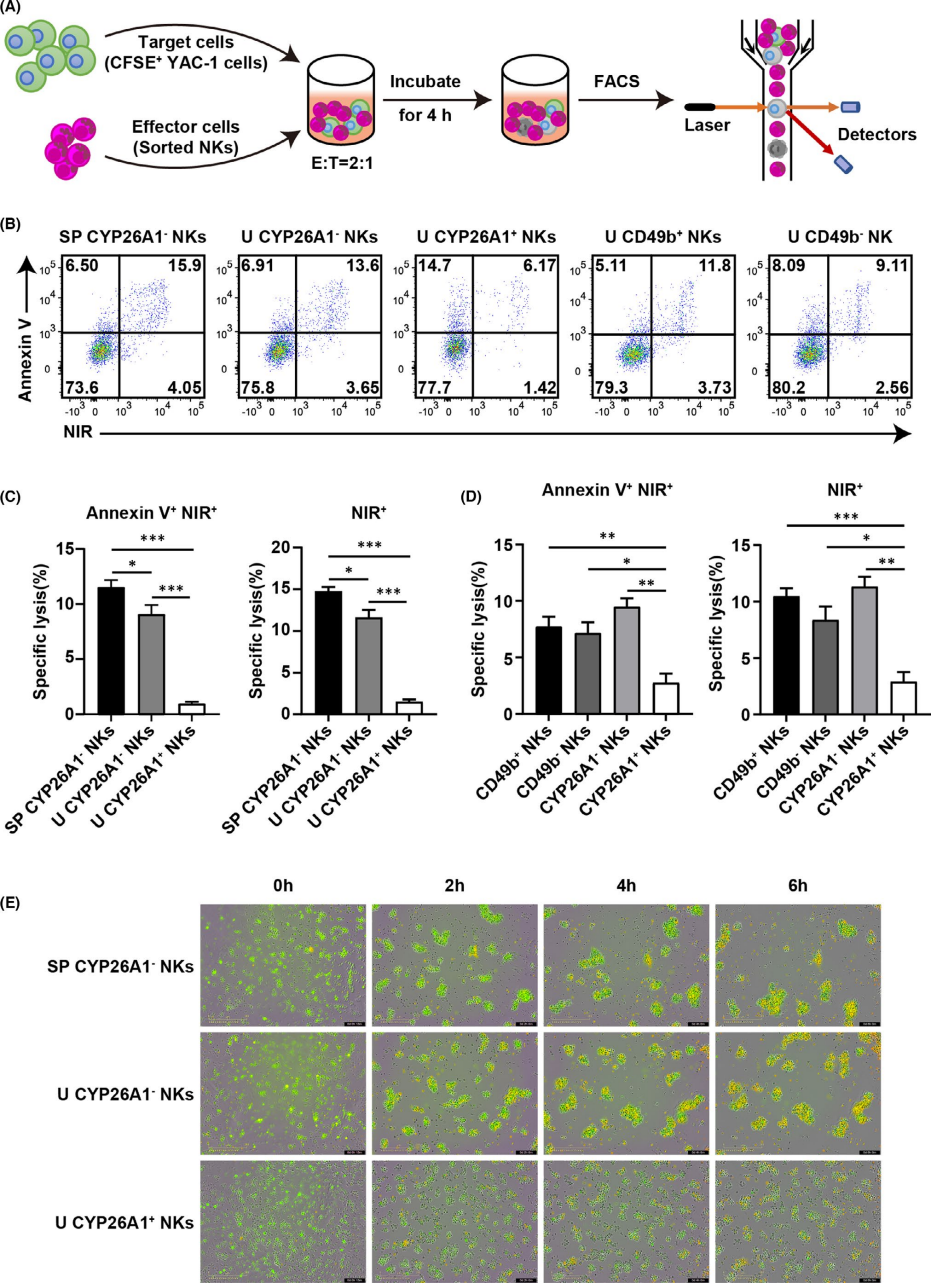
Uterine CYP26A1^+^ NK cells on GD6 had low cytotoxicity. (A) Flowchart of NK cell‐mediated cytotoxicity assay. (B) Representative flow cytometry analysis evaluating lytic activity of splenic and uterine NK cells. YAC‐1 cells were labelled with CFSE and co‐cultured with NK cells at an E:T ratio of 2:1. After 4 h, all samples were stained with Annexin V‐PE and Helix NP™ NIR and then analysed by flow cytometry. Statistical analysis of percentages of Annexin V^+^NIR^+^ and NIR^+^ cells among YAC‐1 cells killed by (C) splenic CYP26A1^−^ cells and uterine CYP26A1^−^ and CYP26A1^+^ cells, and (D) CD49b^+^, CD49b^−^, CYP26A1^−^, and CYP26A1^+^ NK cells in mice. Unpaired t test. (E) Live cell imaging of NK cells killing YAC‐1 cells at different time points. YAC‐1 cells were marked with CFSE (green); dead cells were labelled with propidium iodide (red); dead YAC‐1 cells were stained yellow. Scale bar: 200 µm. NKs, NK cells; SP, spleen; U, uterus. Data represent means ± SEM of at least three independent experiments. *p < 0.05, **p < 0.01, ***p < 0.001

To investigate the function of CYP26A1^+^ NK cells, we performed a flow cytometry‐based cytotoxicity assay to evaluate the killing activity of freshly isolated splenic and uterine NK cells on GD6. Briefly, YAC‐1 targets were labelled with CFSE and then co‐cultured with effector NK cells at an E:T ratio of 2:1 for 4 h (Figure 4A). In the assay, non‐viable apoptotic and dead cells were marked as Annexin V^+^NIR^+^ and NIR^+^ cells respectively (Figure 4B). Uterine CYP26A1^+^ NK cells showed the lowest percentage of non‐viable apoptotic and dead cells within YAC‐1 cells, followed by uterine and splenic CYP26A1^−^ cells (Figure 4C). Further experiments were conducted to assess the lytic capacity of uterine CD49b^+^, CD49b^−^, CYP26A1^−^ and CYP26A1^+^ NK cells. The results indicated that CYP26A1^+^ NK cells had lower killing capacity than the other three uterine NK cell subsets (Figure 4D). We also monitored cell viability in real‐time using an Incucyte S3 live‐cell analysis system. YAC‐1 cells co‐cultured with uterine CYP26A1^+^ NK cells showed a higher viability than CYP26A1^−^ NK cells from the spleens and uteri of pregnant mice (Figure 4E). These results demonstrated that uterine CYP26A1^+^ NK cells had low killing activity during early pregnancy.

### 3.5 CYP26A1^+^ NK cells with specifical transcriptional profile

**FIGURE 5:**
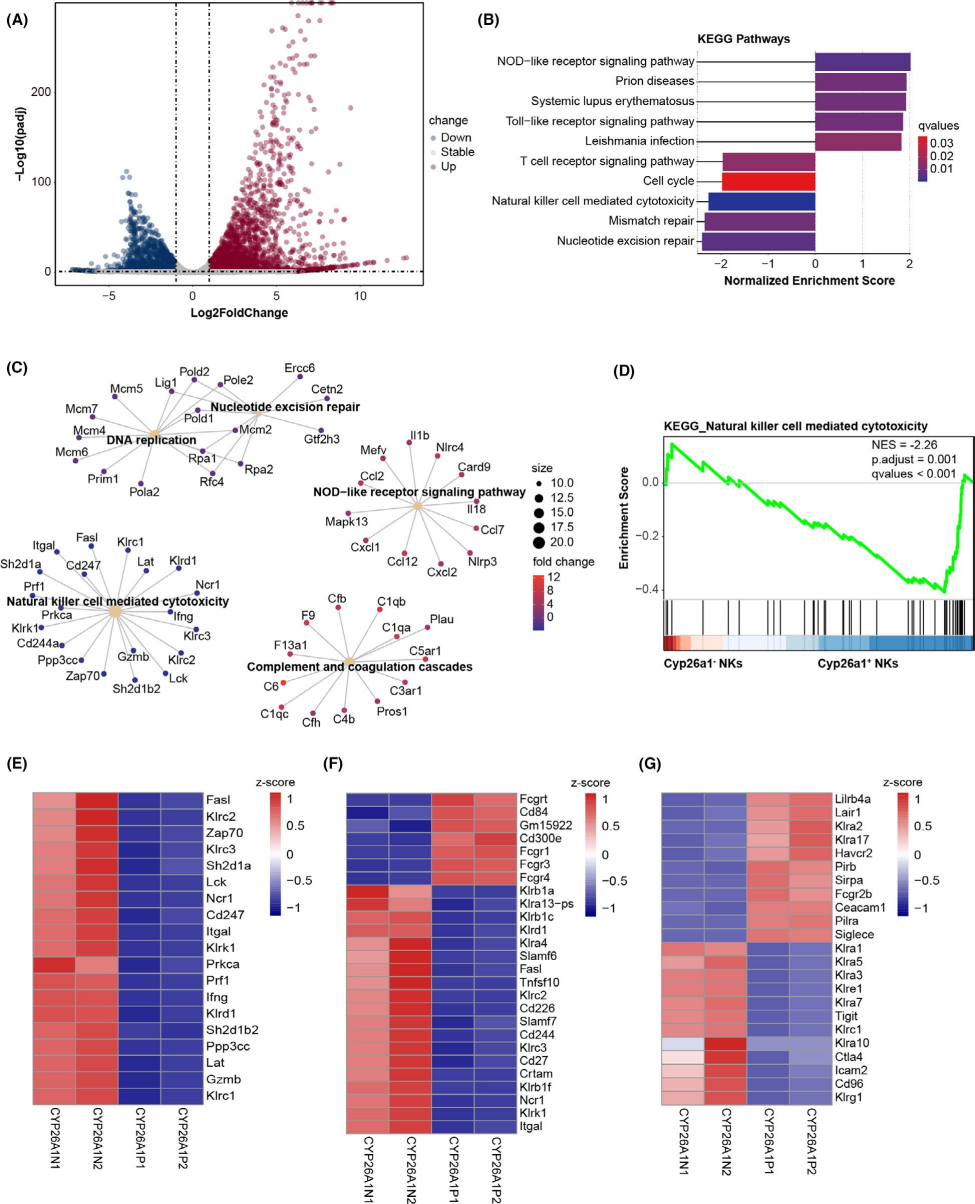
GSEA analysis for RNA sequencing of uterine CYP26A1^−^ and CYP26A1^+^ NK cells at GD5. (A) Volcano plot describing differentially expressed genes. Blue and red dots represent genes with lower and higher expression levels in CYP26A1^+^ NK cells, respectively. (B) Bar plot of GSEA analysis showing 10 enriched KEGG pathways. The adjusted *p* values of these 10 pathways were all <0.05. If the normalized enrichment score (NES) was greater than 0, the pathway was upregulated; otherwise, it was downregulated. These 10 pathways represented the top 5 upregulated and downregulated pathways ordered by the absolute value of NES. q‐values indicate the false discovery rate. (C) cnetplot depicting gene overlap relationship among top 5 KEGG pathways ordered by adjusted *p* value. The adjusted *p* values of these 5 pathways were all <0.05. (D) Enrichment plot for the natural killer cell mediated cytotoxicity pathway. (E) Heatmap showing expression of core enrichment genes in gene set shown in (D). Z‐scores of expression of (F) activating receptors and (G) inhibitory receptors. CYP26A1N1 and CYP26A1N2 denote CYP26A1^−^ NK cells; CYP26A1P1 and CYP26A1P2 indicate CYP26A1^+^ NK cells. NKs, NK cells

To further investigate functional differences between uterine CYP26A1^−^ and CYP26A1^+^ NK cells in mice during the peri‐implantation period, we conducted comprehensive transcriptome‐wide screening to evaluate their transcriptional expression profiles. Transcriptome analysis revealed 4612 differentially expressed genes (adjusted *p* < 0.05; |Fold change| > 2). Among them, 1,669 genes displayed lower expression, whereas 2,943 genes were upregulated in CYP26A1^+^ NK cells (Figure 5A). We then used these differentially expressed genes to perform GSEA. The 10 KEGG pathways significantly enriched in GSEA are shown in Figure 5B. We also performed gene overlap relationship analysis on the top 5 pathways enriched by KEGG (Figure 5C). Interestingly, the natural killer cell‐mediated cytotoxicity pathway was enriched with an adjusted *p* value of 0.001 (Figure 5B,C). The pathway was dramatically downregulated in CYP26A1^+^ NK cells (Figure 5D). Compared with CYP26A1^−^ NK cells, CYP26A1^+^ NK cells displayed lower expression levels of pivotal genes involved in this pathway, such as *Prf1*, *Gzmb* and *Fasl* (Figure 5E). Activating and inhibitory receptors also modulated NK cell activity. We found that the majority of activating receptors, such as *Cd226*, *Klrk1 and Ncr1* had lower expression levels in CYP26A1^+^ NK cells (Figure 5F). The levels of inhibitory receptors such as *Siglece*, *Fcgr2b* and *Klra2* were higher in CYP26A1^+^ NK cells (Figure 5G).

### 3.6 RNA‐seq data validation

**FIGURE 6:**
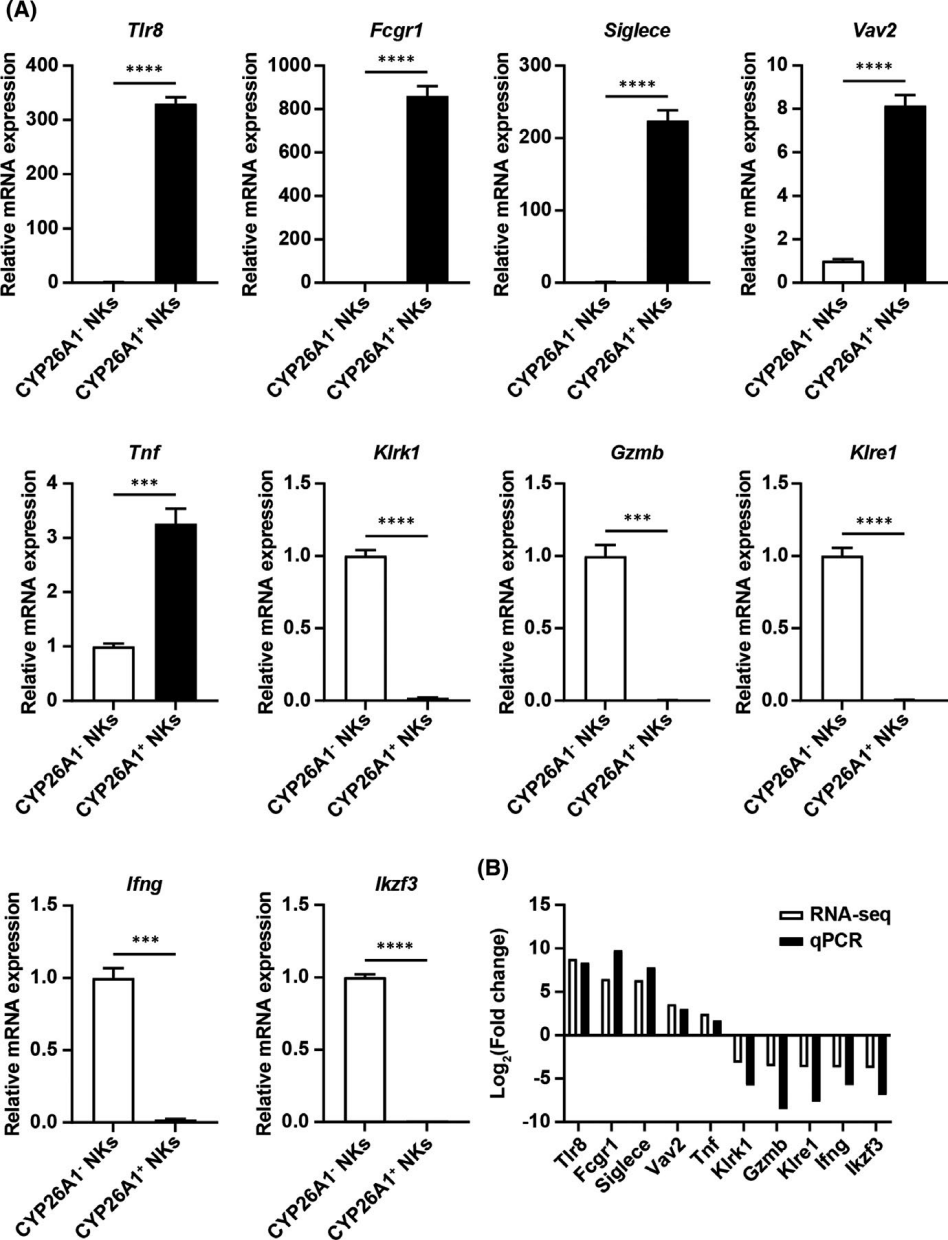
Verification of RNA‐seq data using qPCR. (A) Relative expression of *Tlr8*, *Fcgr1*, *Siglece*, *Vav2*, *Tnf*, *Klrk1*, *Gzmb*, *Klre1*, *Ifng*, and *Ikzf3* in uterine CYP26A1^−^ and CYP26A1^+^ NK cells in mice on GD6. (B) Comparison of RNA‐seq and qPCR assay data of selected genes. NKs, NK cells. Data represent means ± SEM of at least three independent experiments. ***p < 0.001, ****p < 0.0001

Using GSEA, we found that the natural killer cell‐mediated cytotoxicity pathway was downregulated in CYP26A1^+^ NK cells, and that these cells had a very specific receptor repertoire. To verify the accuracy of the transcriptome sequencing results, qPCR was used to detect the expression of the following genes in CYP26A1^−^ and CYP26A1^+^ NK cells from the uteri of pregnant mice on GD6: (i) *Tlr8* and *Ikzf3* (randomly chosen); (ii) *Vav2*, *Tnf*, *Gzmb* and *Ifng* (associated with the natural killer cell mediated cytotoxicity pathway); (iii) *Fcgr1* and *Klrk1* (activating receptors); (iv) *Siglece* and *Klre1* (inhibitory receptors). The qPCR results were consistent with the RNA‐seq data (Figure 6A,B). Our transcriptome sequencing results are hence highly credible and can act as evidence for the low killing activity of CYP26A1^+^ NK cells.

## 4 DISCUSSION

CYP26A1, an RA‐metabolizing enzyme, has already been verified to play a prominent role in embryo implantation. Both inhibition and blockade of CYP26A1 function results in a significant reduction in the number of embryo implantation sites in mice.^19^ Further studies have indicated that the at‐RA concentration in the uterus has no marked difference after CYP26A1 knockdown (data not published) and that intraperitoneal administration of supraphysiological doses of at‐RA has no influence on embryo implantation in mice.^23^ These results indicate that CYP26A1 may regulate embryonic implantation via a non‐RA pathway. Recently, we found that CYP26A1 regulates the differentiation of DCs (through CD86 and ID2), polarization of uterine macrophages, and proportion of NK cells during the peri‐implantation period in mice.^21^, ^22^, ^23^ We hence conclude that CYP26A1 affects embryo implantation through immune cells at the maternal‐foetal interface.

To the best of our knowledge, this study is the first to provide a single‐cell transcriptomics atlas of NK cells at the maternal‐foetal interface in Cyp26a1‐MO knockdown mice. We defined four major subsets of uterine NK cells: cNK, trNKp, trNK1 and trNK2 cells. CYP26A1 knockdown did not change the clusters of uterine NK cells but affected their proportion. We found that Cyp26a1‐MO knockdown mice harboured a lower ratio of trNKp cells and an increased proportion of trNK2 cells compared with the controls. Blastocysts are implanted successfully into the receptive endometrium through apposition, attachment and penetration, which involves a series of events such as decidualization and angiogenesis.^31^, ^32^, ^33^ VEGF‐C accelerates immune tolerance of uterine NK cells by inducing TAP‐1, and facilitates active angiogenesis during early gestation.^11^, ^34^ CD44 and CD160 are activation markers of NK cells.^30^ CYP26A1 knockdown significantly downregulated the expression of the inhibitory receptor *Klra17* and the growth‐promoting factor *Ogn* in trNKp cells, as well as that of *Vegfc*, *Cd44*, *Cd160*, and the activating receptor *Slamf6* in trNK2 cells. In addition, the mRNA expression levels of cytokines and chemokines in mouse uterus changed significantly after CYP26A1 knockdown, which may be closely related to the change in the proportion of NK cell subsets and their immune activity.^23^ However, the exact mechanism needs to be clarified through further experiments.

Our laboratory has found that there is a population of CYP26A1^+^ NK cells at the maternal‐foetal interface.^25^ Unfortunately, we detected only five NK cells expressing *Cyp26a1* via scRNA‐seq (data not shown). The most likely explanation is that the latter had limited sequencing depth. Flow cytometry and immunofluorescence assays confirmed that the CYP26A1^+^ NK cell subset specifically existed in the uteri of pregnant mice, which dovetails nicely with the results for humans.^24^, ^35^ This NK cell subset exhibited dynamic changes during the peri‐implantation period. Cytotoxicity assays demonstrated that CYP26A1^+^ NK cells had low killing activity. Given that NK cell activity could be modulated by inhibitory and activating surface receptors,^36^, ^37^, ^38^, ^39^, ^40^ RNA‐seq was performed to determine the transcriptional profile of CYP26A1^+^ NK cells. RNA‐seq analysis revealed that the NK cell‐mediated cytotoxicity pathway and most of the activated receptors were significantly downregulated in CYP26A1^+^ NK cells. These results demonstrated that CYP26A1^+^ NK cells had low killing capacity.

In conclusion, our data indicated that CYP26A1 knockdown had no effect on the clusters of NK cells in the uterus, but it altered the proportion of NK cell subsets and significantly down‐regulated the expression levels of cytokines and immunologic activity‐related genes in NK cells. CYP26A1^+^ NK cells exhibited low killing activity. CYP26A1 may affect the immune microenvironment at the maternal‐foetal interface by regulating the activity of NK cells.

## CONFLICT OF INTEREST

The authors confirm that there are no conflicts of interest.

## AUTHOR CONTRIBUTIONS

**Dan‐Dan Li:** Conceptualization (lead); Data curation (lead); Investigation (lead); Methodology (lead); Visualization (lead); Writing – original draft (lead); Writing – review & editing (lead). **Wen‐Heng Ji:** Investigation (equal); Methodology (supporting); Visualization (equal); Writing – review & editing (equal). **Dan‐Ping Wei:** Investigation (equal); Methodology (supporting). **Ai‐Qin Gu:** Methodology (supporting). **Zhi‐Hui Song:** Methodology (supporting). **Wen‐Ning Fang:** Methodology (supporting). **Chao‐Yang Meng:** Methodology (supporting). **Ying Yang:** Methodology (supporting). **Jing Pian Peng:** Funding acquisition (lead); Methodology (supporting); Resources (lead); Supervision (lead); Writing – review & editing (equal).

## Data Availability

We confirm that the data in our paper can be used.
